# Removal of EOG Artifacts from EEG Recordings Using Stationary Subspace Analysis

**DOI:** 10.1155/2014/259121

**Published:** 2014-01-12

**Authors:** Hong Zeng, Aiguo Song

**Affiliations:** School of Instrument Science and Engineering, Southeast University, Nanjing 210096, China

## Abstract

An effective approach is proposed in this paper to remove ocular artifacts from the raw EEG recording. The proposed approach first conducts the blind source separation on the raw EEG recording by the stationary subspace analysis (SSA) algorithm. Unlike the classic blind source separation algorithms, SSA is explicitly tailored to the understanding of distribution changes, where both the mean and the covariance matrix are taken into account. In addition, neither independency nor uncorrelation is required among the sources by SSA. Thereby, it can concentrate artifacts in fewer components than the representative blind source separation methods. Next, the components that are determined to be related to the ocular artifacts are projected back to be subtracted from EEG signals, producing the clean EEG data eventually. The experimental results on both the artificially contaminated EEG data and real EEG data have demonstrated the effectiveness of the proposed method, in particular for the cases where limited number of electrodes are used for the recording, as well as when the artifact contaminated signal is highly nonstationary and the underlying sources cannot be assumed to be independent or uncorrelated.

## 1. Introduction

The electroencephalographic (EEG) provides a noninvasive facility to investigate the intricacy of human brain. It has been applied in numerous applications such as brain-computer interface and clinical diagnosis of neurological disorders [1]. A common problem in EEG applications is that the EEG is susceptible to artifacts in the data acquisition [2]. For example, the electric potentials created during eye movement and blinks can be orders of magnitude larger than the EEG and can propagate across much of the scalp, distorting EEG signals. Consequently, such electrooculographic (EOG) artifacts will hinder the interpretation of EEG, it is thereby important to remove the EOG artifacts before further analysis of EEG.

In the literature, the most common EOG artifacts removal method is based on the blind source separation (BSS), usually by independent component analysis (ICA) [3] and second-order blind identification (SOBI) [4]. Such approaches assume that the recorded EEG signals are represented by a limited number of components (or “sources”) and then discarded by those responsible for artifacts during the reconstruction. It has been shown that they are useful in many EOG artifact removal applications [5–8].

However, the classic BSS techniques such as ICA and SOBI may not be effective on the highly nonstationary EOG artifact-contaminated EEG recordings. On one hand, the ocular artifacts resulting from eye movement and blink demonstrate strongly nonstationary characteristics within a considerably long interval: it is often localized with abruptly large amplitude and low frequency; its duration and amplitude appear to differ stochastically and considerably between successive eye movements or blinks. This implies that there are significant distribution changes in the artifact-contaminated EEG observations, such as the changes in the mean and the covariance matrix. However, ICA is not devoted to the understanding of the distribution changes but to find the components that are both statistically independent and non-Gaussian [3]. Although SOBI exploits the temporal changes in the covariance matrix of the observations by the joint diagonalization of several covariance matrices with different time delays, the changes in the mean of the observations have not been taken into account. On the other hand, the underlying sources associated with artifacts may not be assumed to be independent or uncorrelated among each other. It is known that ICA and SOBI can perform well on the eye blink artifacts contaminated EEG signals [5–8]. This is because blink artifacts mainly involve the vertical movement, the sources corresponding to the vertical movement and horizontal movement of eyes; thus, can be assumed to be independent or uncorrelated. However, when both the vertical and horizontal eye movements are involved in the contamination of the EEG observations, the sources associated with such EOG artifacts cannot be assumed to be independent nor uncorrelated anymore, because the vertical and horizontal eye movements are often accompanied with each other, in particular for eye ball rolling, forming two dependent components. Nevertheless, ICA instead has imposed the requirement of sourcewise independency, while SOBI is based on the assumption that the sources should be uncorrelated with each other. Thus, on the highly nonstationary EOG artifact-contaminated EEG recordings, they raise the problem of “misallocation of variance” [9, 10] and result in suboptimal separation: the artifacts fail to be concentrated in a small number of components by ICA and SOBI. Since it is usually assumed that the number of sources is no greater than the number of channels in the BSS literature, in cases where there are limited number of electrodes used for EEG recording (e.g., often used in sleep studies [11] and when subjects are neonates or young infants, due to the size of the head [12]), it may lead to the loss of information related to brain activity by rejecting those sources found by ICA or SOBI, as such sources generally also contain neural activity aside from pure artifacts [13, 14].

To addresses the weakness of ICA-based and SOBI-based artifact correction methods on highly nonstationary EEG recordings with limited number of electrodes, this paper proposes a novel EOG artifact removal approach that utilizes the recently proposed effective BSS method, that is, stationary subspace analysis (SSA) [15, 16]. Unlike the classic blind source separation with ICA or SOBI, the adopted BSS algorithm SSA is explicitly tailored to the understanding of distribution changes [16]. The type of distribution changes that SSA detects is changes in both the mean and the covariance matrix. In addition, neither independency nor uncorrelation is required among the sources by SSA. Subsequently, artifacts can be concentrated in only a few components. To the best of our knowledge, SSA has not been applied to EEG signals for removing artifacts thus far, even though it has been shown to have interesting applications in robust motor imagery prediction for Brain-Computer Interface [15, 17], geophysical data analysis [16], WiFi localisation [18], computer vision [19], and change-point detection [20]. Experiments on both simulated data and real EEG recordings are conducted, and the results show that the proposed method can effectively improve the artifact correction on raw EEG recordings.

The remainder of the paper is organized as follows. In Section 2, we present the proposed approach for EOG artifact correction in detail. Experimental results are given in Section 3. Finally, conclusions and discussion are presented in Section 4.

## 2. Proposed Approach for EOG Artifact Removal

**Figure 1:**
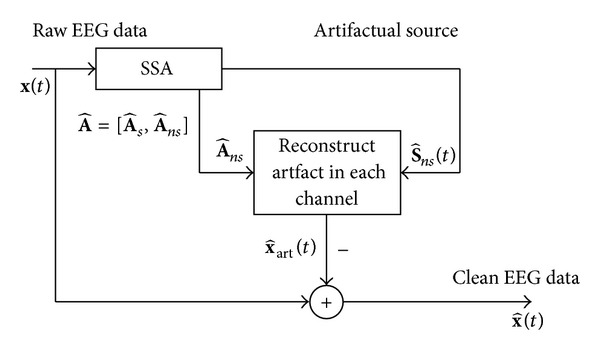
Block diagram of the proposed approach.

Figure 1 shows the block diagram of the proposed approach. It comprises the following key steps: (1) apply SSA to obtain the components containing artifacts ${\hat{S}}_{ns}(t)$, (2) reconstruct the EOG artifact at the scalp electrodes ${\hat{x}}_{\text{art}}(t)$ with the artifactual components ${\hat{S}}_{ns}(t)$, and (3) subtract the estimated artifacts ${\hat{x}}_{\text{art}}(t)$ from the raw EEG recordings to get clean EEG $\hat{x}(t)$.

### 2.1. Blind Source Separation by SSA

The first key step in the proposed approach is the application of SSA to separate the artifactual components from the raw EEG data. The observed signal **x**(*t*) is modeled as a linear superposition of two groups of sources with a time-constant invertible mixing matrix **A**. One group includes the sources **S**_*s*_(*t*) related to the cerebral activity, the other includes the sources **S**_*ns*_(*t*) that arise from the eye movement and blink, that is, the highly nonstationary EOG artifactual sources whose distribution changes are the most pronounced as follows:

(1) $$\begin{array}{c} x\left(t\right)=AS\left(t\right)=\left[\begin{array}{cc} A_{s} & A_{ns} \end{array}\right]\left[\begin{array}{c} S_{s}\left(t\right)\\ S_{ns}\left(t\right) \end{array}\right], \end{array}$$

where **A**_*s*_ and **A**_*ns*_ are the corresponding mixing matrices for **S**_*s*_(*t*) and **S**_*ns*_(*t*), respectively. To factorize the observed time series **x**(*t*) into the cerebral sources **S**_*s*_(*t*) and the EOG artifactual sources **S**_*ns*_(*t*), SSA is applied to estimate the inverse mixing matrix **A** ^−1^ as $B={\left[\begin{array}{cc} B_{s}^{T} & B_{ns}^{T} \end{array}\right]}^{T}$, such that ${\hat{S}}_{s}(t)={\hat{B}}_{s}x(t)$ and ${\hat{S}}_{ns}(t)={\hat{B}}_{ns}x(t)$ are the estimated cerebral and artifactual sources, respectively. The “weak nonstationarity” criterion is adopted in SSA, that is, the data are split into *N* consecutive epochs, and components are considered to be nonstationary if their empirical distribution (approximated by mean and covariance) changes significantly in these epochs [15, 16]. It has been shown that such a criterion can be approximated as a generalized eigenvalue problem [16]:

(2) $$\begin{array}{c} \underset{B}{\min}\;\mathrm{Tr}\left[BVB^{T}\right]\;\text{subject to}\;\;B\overset{-}{\Sigma }B^{T}=I \end{array}$$

with the matrix **V** defined by

(3) $$\begin{array}{c} V=\frac{1}{N}\sum_{k=1}^{N}\left\{\mu_{k}\mu_{k}^{T}+2\Sigma_{k}\overset{-}{\Sigma }\Sigma_{k}^{T}\right\}-\overset{-}{\mu }{\overset{-}{\mu }}_{}^{T}-2\overset{-}{\Sigma }, \end{array}$$

where *Tr*[·] denotes the trace of a matrix, *T* is the transposition operation, **I** is the identity matrix, ***μ***_*k*_ and Σ_*k*_ are the mean and covariance in the *k*th epoch, respectively, $\overset{-}{\mu }=(1/N)\sum_{k=1}^{N}\mu_{k}$, and $\overset{-}{\Sigma }=(1/N)\sum_{k=1}^{N}\Sigma_{k}$. Such an objective function in (3) essentially aims to minimize the distance between each epoch mean and covariance and their respective averages. This distance is measured using the variance of the mean and covariance across all epochs. This eigen-decomposition problem can be solved efficiently, and then the mixing matrix $\hat{A}$ is obtained by ${\hat{B}}^{-1}$, and the estimated components are derived by

(4) $$\begin{array}{c} \hat{S}\left(t\right)=\hat{B}x\left(t\right). \end{array}$$

As can be observed from (2) and (3), there are two advantages of SSA over ICA and SOBI. Firstly, it explicitly exploits the nonstationarity between epochs, where the distribution changes in both the mean and the covariance matrix have been taken into account. Secondly, it does not require the sourcewise independency or uncorrelation. In fact, SSA allows arbitrary dependence structure among and between the two groups of sources [16]. Therefore, the highly nonstationary eye movement or blink artifacts generally will not spread out over multiple components but be concentrated into only a few components by SSA. Furthermore, SSA orders the components in increasing degree of non-stationarity [16], thereby the components that are likely to be associated with the artifacts would be ranked in the bottom. To automatically identify the artifactual sources ${\hat{S}}_{ns}(t)$, we adopted the effective methods developed in [8].

### 2.2. Reconstruct the EEG Signals

Using the estimated mixing matrix, ${\hat{A}}_{}=[{\hat{A}}_{s}\;\;{\hat{A}}_{ns}]$, given by SSA, these artifactual components ${\hat{S}}_{\text{art}}(t)$ are projected back to EEG channel, and artifacts in EEG data ${\hat{x}}_{\text{art}}(t)$ are calculated as

(5) $$\begin{array}{c} {\hat{x}}_{\text{art}}\left(t\right)={\hat{A}}_{ns}{\hat{S}}_{\text{art}}\left(t\right). \end{array}$$

Finally, this ocular activity is removed from the EEG recording to yield the clean EEG data $\hat{x}(t)$ by

(6) $$\begin{array}{c} \hat{x}\left(t\right)=x\left(t\right)-{\hat{x}}_{\text{art}}\left(t\right). \end{array}$$

The complete steps of the proposed approach are summarized as follows. 

*Summary of Proposed Approach for EOG Artifacts Correction from Raw EEG Recordings*

*Processing:*

1. apply SSA on *x*(*t*), and then identify the artifactual components ${\hat{S}}_{ns}(t)$ as well as the corresponding columns of mixing matrix ${\hat{A}}_{ns}$;
2. estimate the artifacts in multichannel EEG data by ${\hat{x}}_{\text{art}}(t)={\hat{A}}_{ns}{\hat{S}}_{\text{art}}(t)$;
3. subtract the artifacts from EEG data to get clean EEG: $\hat{x}(t)=x(t)-{\hat{x}}_{\text{art}}(t)$.

## 3. Experiments

### 3.1. Suppression of Artifact on the Artificially Contaminated EEG Signals

#### 3.1.1. Data Generation

Forty healthy volunteers, 20 males and 20 females, aged between 20 and 33 years (mean age 27.6 years) were involved in the study.

**Figure 2:**
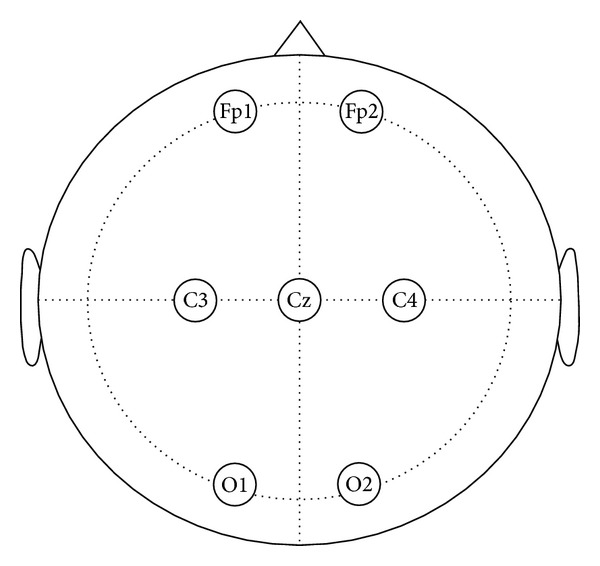
Placement of the EEG electrodes on the scalp according to the recording 10–20 system.

The EEG signals were recorded on 20 volunteers with the NeuroScan SynAmps2 system. 6 EEG channels (Fp1, Fp2, C3, C4, O1, O2) were used for recording and the ground electrode was placed at position Cz, according to the 10–20 system (Figure 2). To obtain the pure EEG data to be artificially contaminated, a 50s consecutive epoch, where there is no obvious artifacts with a careful inspection, was recorded for each volunteer in an eyes-closed session. Signals were digitized at a rate of 250 Hz, band pass filtered at 0.5–50 Hz, and notch filtered at 50 Hz.

To obtain the artifact signals for contaminating the pure EEG, separate EOG signals were obtained on the remaining 20 volunteers during eyes-open sessions with eye rolling, which were recorded by two electrodes placed above and below the left eye and another two on the outer canthi. This process gave rise to two bipolar signals for each volunteer, namely, vertical-EOG (VEOG), which is equal to the upper minus lower EOG electrode recordings and horizontal-EOG (HEOG), which is equal to the left minus right EOG electrode recordings. These EOG signals were band pass filtered between 0.5 and 15 Hz.

Finally, to generate the “artificially contaminated EEG signals” (used for evaluating the performance of approaches for EOG artifact correction), we have used the Elbert's contamination model [21]:

(7) $$\begin{array}{c} \text{mixe}{\text{d}}_{j}=\text{inEE}{\text{G}}_{j}+\alpha_{j}\text{VEOG}+\beta_{j}\text{HEOG}, \end{array}$$

where mixed_*j*_ is the artificially contaminated EEG signal on the *j*th channel and inEEG_*j*_ is the collected artifact-free EEG signal described above. Variables *α*_*j*_, *β*_*j*_ denote the propagation weights for VEOG and HEOG, respectively, initialized according to [22]. The mixing procedure by means of (10) was carried out 20 times in order to simulate 20 subjects or sets of mixed signals.

**Figure 3:**
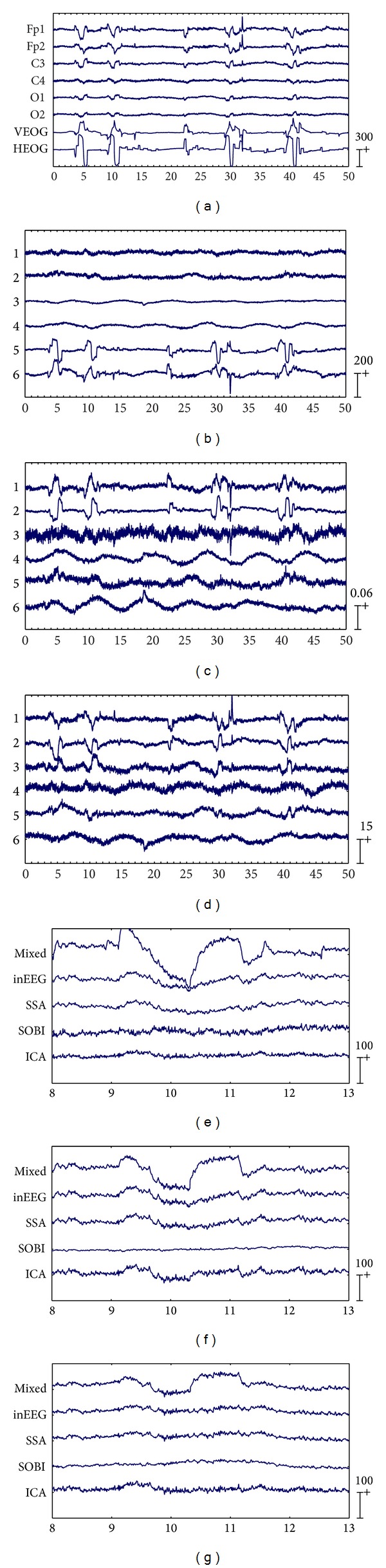
Results of EOG artifact removal on an example EEG data set from the 20 artificially contaminated ones. The artificially contaminated EEG data with eye movement and blink artifacts are shown in the first six EEG channels of (a) and the corresponding EOG signals used in the mixing procedure are presented in the last two channels of (a). Components separated by SSA, SOBI, and ICA are shown in (b), (c), and (d), respectively. The zoomed mixed, precontaminated EEG, and corrected EEG signals by each method on channel Fp1, C3, and O1 are depicted in (e), (f), and (g), respectively.

The first six channels in Figure 3(a) depict an example from one of the 20 artificially contaminated EEG signals, while the last two channels show the corresponding VEOG and HEOG used to generate the mixed signal. The eye movement and blink artifacts appear in EEG as big pulses localized in time and have a strong impact to EEG signals. Besides, the eye movement and blink episodes are spread over all channels and affect most strongly the frontal sites (Fp1, Fp2).

#### 3.1.2. Performance Measures

Thanks to the precontaminated EEG signals described above, we were able to conduct quantitative comparison between the original and the corrected signals. Two commonly used evaluation metrics were adopted in the experiments with two goals to test the quality of recovering the cerebral signals and the degree of removing the ocular artifacts.

(*1) Mutual Information.* The mutual information (MI) quantifies the mutual dependence of the precontaminated EEG signals and the artifact-corrected EEG data sets using the following formula [14]:

(8) MI(inEEG,outEEG)  =∑x∈inEEG ∑y∈outEEGp(x,y)log(p(x,y)p1(x)p2(y)),

where the inEEG denotes the artifact-free EEG data set before the contamination (Section 3.1.1) and the outEEG stands for the cleaned EEG signals by an approach for artifact correction. *p*(*x*, *y*) is the joint probability distribution function, and *p*_1_(*x*) and *p*_2_(*y*) are the marginal probability distribution functions of inEEG and outEEG, respectively. The larger the MI, the better the corrected EEG resembles the original EEG. We used this metric to quantify the quality of the recovered cerebral signals.

(*2) Improvement of Signal-to-Artifact Ratio.* The initial signal-to-artifact ratio (SAR) before the artifact correction was computed for each EEG channel in order to describe the extent of ocular contamination over the scalp. It was defined as follows:

(9) $$\begin{array}{c} \text{SA}{\text{R}}_{\text{before}}=10\log\frac{\text{Energy}\left\{\text{inEEG}\right\}}{\text{MSE}\left\{\text{mixed}-\text{inEEG}\right\}}. \end{array}$$

After the artifact correction, the final SAR was calculated by

(10) $$\begin{array}{c} \text{SA}{\text{R}}_{\text{after}}=10\log\frac{\text{Energy}\left\{\text{inEEG}\right\}}{\text{MSE}\left\{\text{outEEG}-\text{inEEG}\right\}}. \end{array}$$

Then the improvement of SAR (ΔSAR) was obtained by subtracting the final SAR from the initial SAR [10] as follows:

(11) $$\begin{array}{c} \Delta \text{SA}{\text{R}}_{}=10\log\frac{\text{MSE}\left\{\text{mixed}-\text{inEEG}\right\}}{\text{MSE}\left\{\text{outEEG}-\text{inEEG}\right\}}. \end{array}$$

An effective correction method would result in a good outEEG that is almost the same as the inEEG, thereby MSE between them would be close to zero, consequently leading to a large value for ΔSAR. This metric was utilized to evaluate the degree of removing the ocular artifacts.

#### 3.1.3. Evaluation of Different Artifacts Correction Methods

The proposed SSA-based method (referred to as SSA) was compared with SOBI-based approach (referred to as SOBI) and ICA-based approach (referred to as ICA). For SOBI and ICA, we adopted the SOBI and infomax ICA algorithms implemented in the EEGLAB toolbox [23]. SSA was implemented based on the paper [16]. To automatically identify the artifactual components after performing the blind source separation on the contaminated EEG signal, we adopted the method developed in [8].

By applying SSA on the EEG data on the first six channels shown in Figure 3(a), six components were obtained and depicted in Figure 3(b). The last two components in Figure 3(b) were identified as the artifactual ones by the method in [8], which were strongly nonstationary, featuring abrupt pulses with large amplitude and short-duration. By observing the HEOG and VEOG signals that propagated onto the 6 EEG channels, we found that the last two artifactual components actually reflected the horizontal and vertical motion of eyes, respectively. Finally, the artifactual components were projected back to each channel for estimating the artifacts, and then the corrected EEG data were obtained by subtracting the estimated artifacts from the raw EEG recordings.

For the example data shown in Figure 3(a), the components separated by SOBI and ICA are shown in Figures 3(c) and 3(d), respectively. The first three components by both SOBI and ICA were determined to be responsible for the ocular artifacts according to the method in [8]. In other words, SOBI and ICA failed to capture the vertical and horizontal movements of eyes into two components and the artifacts both spread into the first three components instead.

#### 3.1.4. Evaluation Results

**Figure 4:**
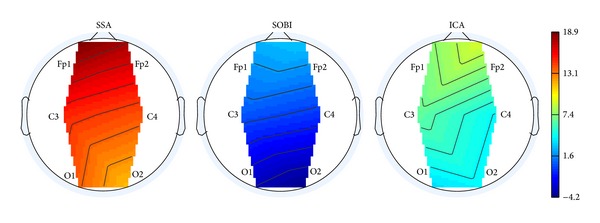
Topographic map showing improvements of SAR after applying the ocular artifact correction procedure.

(*1) Performance of Recovering the Cerebral Signals.* For the example EEG data set shown in Figure 3(a), visual comparison of the zoomed mixed, the precontaminated EEG, and the corrected EEG signals by different ocular artifacts removal methods are given in Figures 3(e), 3(f), and 3(g), where only the EEG signals on the channel Fp1, C3, and O1 are depicted since the result on each of the three right channels is very similar to that on the symmetric left one. The visual inspection confirms that all these three methods effectively suppress the EOG artifacts. However, it can be seen that compared to SOBI and ICA, SSA gives better approximations of the precontaminated EEG signals. Moreover, the mean and standard deviation of MI over 20 simulated EEG data sets, which are reported in Table 1, also tell that SSA outperforms SOBI and ICA, indicating that SSA recovers the cerebral signals from the artificially contaminated EEG recordings most successfully among all the methods.

(*2) Performance of Removing the Ocular Artifacts.* Figure 4 shows the topographic maps corresponding to the average improvements of SAR for each channel, which were obtained by means of the ocular correction methods over the 20 simulated EEG data sets. The mean ΔSAR reached up to 4 dB for fronto-polar channels (Fp1 and Fp2) with the SOBI-based approaches, whereas improvement with ICA techniques was around 9 dB for channel Fp1 and Fp2. SSA provided the highest improvement of SAR (range: from 11.4 dB (O2) to 18.9 dB (Fp1)).

#### 3.1.5. Discussion on the Evaluation Results

There are three important characteristics of the artificially contaminated EEG signals. One is that the EEG data set was recorded with limited number of channels (6 channels, see Section 3.1.1), implying that the BSS algorithms can only separate the signal into 6 or even less underlying sources. Another is that the signal is highly nonstationary within the 50s interval. The last is that the eye rolling artifactual sources used to construct the signal cannot be assumed to be independent or uncorrelated, since the vertical and horizontal movements of eyes were always accompanied with each other when eyes roll. On such data sets, both SOBI and ICA split the artifacts in more than necessary components due to their drawbacks. Consequently, the recovered EEG signals were considerably distorted through rejecting more than necessary number of artifactual components during the reconstruction (see Figures 3(e), 3(f), and 3(g) and Table 1). In contrast, because the distribution changes in both the mean and the covariance matrix have been explicitly taken into account by SSA, and neither sourcewise independency nor uncorrelation is assumed for the underlying sources, SSA succeeded to concentrate the EOG artifacts into fewer components. According to the results on the artificially contaminated EEG recordings, SSA removes the most ocular artifacts from the mixed EEG data and at the same time preserves the most EEG signals.

### 3.2. Suppression of Artifact on the Real EEG Signals

#### 3.2.1. Data Description

**Figure 5:**
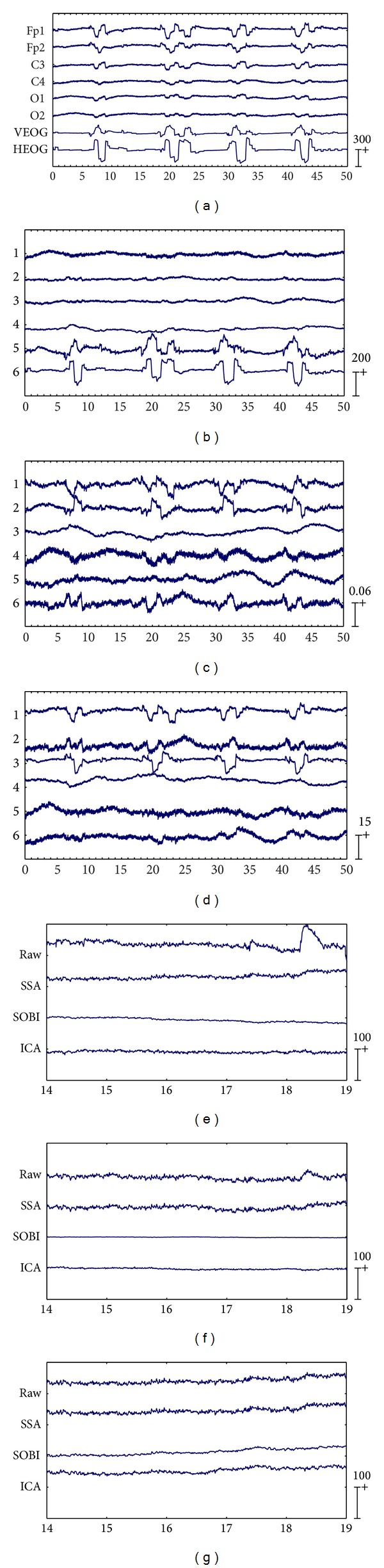
Results of EOG artifact removal on an example EEG data set from the 20 real recordings. The EEG data with eye movement artifacts are shown in the first six EEG channels of (a) and the corresponding EOG signals are presented in the last two channels of (a). Components separated by SSA, SOBI, and ICA are shown in (b), (c), and (d), respectively. The zoomed raw and corrected EEG signals by each method on channel Fp1, C3, and O1 are depicted in (e), (f), and (g), respectively.

In this section, we applied the artifact correction methods on real EEG data sets. Twenty volunteers, 10 males and 10 females, aged between 20 and 32 years old took part in the data collection. The data sets also contain EOG artifacts from rotation movement of the eye balls, which were recorded using the same collection configuration shown in Figure 2. As can be seen from Figure 5(a) which shows an example from the 20 real EEG signals, the EOG artifacts were present on all six EEG channels, while the artifacts are much stronger on the frontal lobe electrodes (Fp1, Fp2) and highly nonstationary (its amplitude differs between successive eye movements).

#### 3.2.2. Evaluation of Different Artifacts Correction Methods

For the EEG data on the first six channels shown in Figure 5(a), the six components separated by SSA are shown in Figure 5(b), where the last two components were identified as the artifactual ones by the method in [8]. These two components accounted for the eye rolling. Thereby, the last two components were projected back to each channel for estimating the artifacts, and then the corrected EEG data were obtained by subtracting the estimated artifacts from the raw EEG recordings.

The results of components separation by SOBI and ICA on the same data are shown in Figures 5(c) and 5(d), respectively. According to the identification results by the method [8], SOBI grasped the time courses of the eye movement artifacts in component 1, 2, 4, and 6, while ICA located the artifacts in the top three components.

#### 3.2.3. Evaluation Results

Figures 5(e), 5(f), and 5(g) demonstrate the visual comparison of the zoomed raw real EEG recording and the zoomed corrected EEG recording by different ocular artifacts correction methods on channel Fp1, C3, and O1 (results on channel Fp2, C4, and O2 are similar to those on Fp1, C3, and O1, thus, are not presented). The EOG artifacts are effectively suppressed by all the three methods. However, it can be observed that SOBI and ICA have introduced significant distortions to the EEG signals. In contrast, as can be seen, SSA has distorted much less EEG signals. To quantitatively evaluate the similarity between the raw EEG signal and the corrected signal within all the artifact-free intervals of the recording, we computed the MI between them. Here the criterion to select an artifact-free segment was that no samples of the VEOG or HEOG exceeded 40 *μ*V. The mean and standard deviation of MI over 20 real EEG recordings are given in Table 2, which have again confirmed that the recovered EEG signals based on SSA best resemble the raw ones during the artifact-free periods.

#### 3.2.4. Discussion on the Evaluation Results

The real EEG recordings have the same three characteristics as the artificially contaminated EEG signals. Both SOBI and ICA again failed to concentrate the artifacts in fewer components again on such kind of data sets. Consequently, the removal of the contaminated components, followed by a signal reconstruction has led to distortions of the underlying cerebral activity. By contrast, SSA captured the eye movement activities in fewer components. Thereby, the proposed method has effectively suppressed the EOG artifacts, while kept the cerebral activities almost intact.

## 4. Conclusions and Discussion

An effective approach is proposed in this paper to address the problem of ocular artifact removal from raw EEG recording. To extract artifactual sources from the raw EEG recording, we have employed the stationary subspace analysis algorithm, which can concentrate artifacts in fewer components than the representative blind source separation methods. Then the artifactual components are projected back to be subtracted from EEG signals, producing the clean EEG data eventually. The experimental results on both the artificially contaminated EEG data and real EEG data have demonstrated the effectiveness of the proposed method, in particular for the cases where limited number of electrodes are used for the recording, as well as when the artifact contaminated signal is highly nonstationary and the underlying sources cannot be assumed to be independent or uncorrelated.

The discussion here is intent to enhance the awareness that BSS algorithms may not produce physical meaningful components unless they take the characteristics of the signals into account and their underlying assumption meets the properties of signals to be analyzed. Unlike the classic blind source separation algorithms, SSA is explicitly tailored to the understanding of distribution changes, where both the mean and the covariance matrix are taken into account. In addition, neither independency nor uncorrelation is required among the sources by SSA. Thereby, it can concentrate artifacts in fewer components than the representative blind source separation methods, leading to better artifacts removal performance in the difficult scenarios mentioned above.

It has been shown that the components selected for removal may also contain neural activity aside from pure artifacts, in particular when there are limited number of recording electrodes [7, 13]. Therefore, our future research will focus on additional methods for postprocessing of the components. Recovering the cerebral signal from the artifactal components via signal decomposition techniques is a possible direction for future research.

## Figures and Tables

**Table 1:** MI by different EOG artifact correction methods over 20 artificially contaminated EEG data sets.

Channel	SOBI	ICA	SSA
Fp1	0.102 ± 0.062	0.169 ± 0.084	0.944 ± 0.105
Fp2	0.096 ± 0.058	0.171 ± 0.063	0.959 ± 0.274
C3	0.041 ± 0.031	0.945 ± 0.306	1.408 ± 0.331
C4	0.078 ± 0.029	0.863 ± 0.265	1.561 ± 0.412
O1	0.608 ± 0.397	0.966 ± 0.411	1.901 ± 0.178
O2	0.446 ± 0.243	1.325 ± 0.386	2.008 ± 0.109

**Table 2:** MI by different EOG artifact correction methods over 20 real EEG data sets.

Channel	SOBI	ICA	SSA
Fp1	0.258 ± 0.156	0.314 ± 0.127	0.763 ± 0.273
Fp2	0.277 ± 0.211	0.318 ± 0.092	0.795 ± 0.346
C3	0.302 ± 0.268	0.390 ± 0.238	1.124 ± 0.463
C4	0.281 ± 0.165	0.426 ± 0.321	1.540 ± 0.107
O1	0.871 ± 0.392	0.989 ± 0.259	1.980 ± 0.215
O2	0.889 ± 0.402	1.376 ± 0.193	2.037 ± 0.134
